# The role of circular RNA in immune response to tuberculosis and its potential as a biomarker and therapeutic target

**DOI:** 10.3389/fimmu.2025.1542686

**Published:** 2025-04-16

**Authors:** Mingyang Hong, Xu Huang, Huiming Zhu, Jiahui Ma, Feng Li

**Affiliations:** ^1^ Department of Clinical Laboratory, Affiliated Nantong Hospital of Shanghai University (The Sixth People’s Hospital of Nantong), Nantong, Jiangsu, China; ^2^ Nantong Institute of Genetics and Reproductive Medicine, Affiliated Maternity and Child Healthcare Hospital of Nantong University, Nantong, Jiangsu, China

**Keywords:** circular RNA, tuberculosis, biomarkers, immune response, therapeutic

## Abstract

Circular RNA (circRNA) is a new type of non-coding RNA that has gained significant attention in recent years, especially in tuberculosis research. Tuberculosis poses a major global public health threat. Its complex pathological mechanisms and worsening drug resistance urgently necessitate new research breakthroughs. The role of circRNA in mycobacterium tuberculosis infection is being gradually revealed, highlighting its importance in regulating gene expression, immune response, and inflammation. Additionally, researchers are interested in circRNA because of its potential for early tuberculosis diagnosis and its role as a biomarker. This article systematically analyzes existing literature to provide new insights into early tuberculosis diagnosis and personalized treatment. We also emphasize the need for future research to enhance the application of circRNA in tuberculosis prevention and control.

## 1 Introduction

Tuberculosis (TB) is a serious infectious disease caused by Mycobacterium tuberculosis (Mtb) and leads to about 1.5 million deaths worldwide every year (1). Although there have been substantial advancements in modern medicine to prevent and treat tuberculosis, the disease continues to be a significant public health challenge globally (2, 3). This is particularly true in developing countries, where high tuberculosis incidence and mortality rates significantly affect social and economic systems (2, 4). Therefore, it is crucial to develop more effective diagnostic and therapeutic tools (5).

Circular RNA (circRNA) is a new type of non-coding RNA characterized by its unique circular shape, which is found throughout eukaryotic cells (6). In contrast to linear RNA, circRNA demonstrates enhanced stability and tissue specificity (7). Recent studies have clarified the role of this factor in various physiological and pathological processes (8). CircRNA is crucial for both tumorigenesis and cardiovascular diseases, and it also plays a role in tuberculosis mechanisms (9, 10). Studies indicate that circRNA can influence both the host immune response and the infection process of pathogens by regulating gene expression, sequestering microRNAs, and interacting with RNA-binding proteins (11, 12). Studies show that circRNA is an important regulator of the immune response against tuberculosis in the host (13). For example, circRNA helps decrease the death of monocytes, boosts the process of autophagy, and aids in the polarization of macrophages, thereby strengthening the host’s ability to resist Mycobacterium tuberculosis (14, 15). In addition to its stability and abundance in various body fluids, circRNA is recognized as a potential biomarker, which positions it as a promising non-invasive diagnostic tool for tuberculosis (16, 17).

Although circRNA shows great potential in TB research, there are still some shortcomings in current studies. For example, the specific mechanisms of circRNA in tuberculosis are not yet fully understood, and the application of circRNA as a diagnostic and therapeutic target still requires further validation (18). Therefore, in-depth research on the biological functions and mechanisms of circRNA in tuberculosis is of significant importance for developing new diagnostic and therapeutic strategies.

In summary, circRNA plays an important role in the pathological mechanisms, early diagnosis, and potential treatment of tuberculosis. This article will review the research progress of circRNA in tuberculosis, exploring its biological functions in Mycobacterium tuberculosis infection, its relationship with immune responses, its potential as a biomarker, and its application prospects in future therapeutic strategies. By analyzing the latest research findings, we hope to provide new insights for the early diagnosis and personalized treatment of tuberculosis.

## 2 Formation and function of circular RNA

### 2.1 Biogenesis mechanism of circRNA

**Figure 1:**
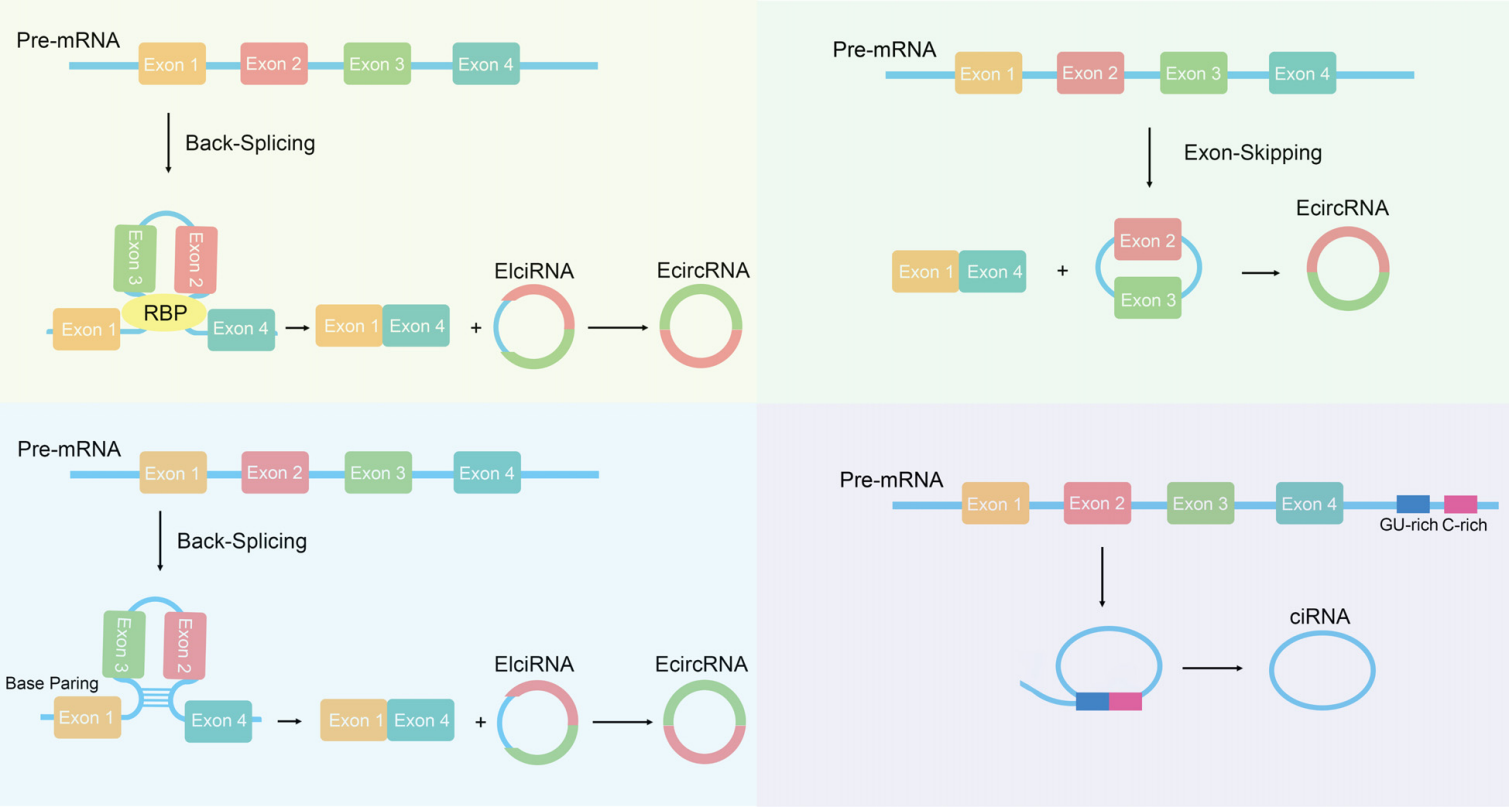
The biogenesis of circRNAs. Exon circular RNA (EcircRNA) can be generated through back-splicing, which is regulated by base pairing between reverse complementary sequences and RNA binding proteins (RBPs). EcircRNAs can also be formed through lariat precursor generated by exon skipping. The generation of Circular intronic RNAs (ciRNAs) depends on the presence of GU-rich sequences and C-rich sequences to form lariat introns, which are then subjected to tail trimming.

CircRNA is a special type of non-coding RNA characterized by the formation of a closed circular structure (19). Compared to linear RNA, circRNA exhibits higher stability and persistence within cells (20). Based on their origin, circRNAs can be divided into three categories: the first category consists of circRNAs made up of exon sequences, known as exonic circRNA (EcircRNA); the second category consists of circRNAs made up of intron sequences, known as intronic circRNA (ciRNA); the third category consists of circRNAs composed of both exons and introns, known as exon-intron circRNA (EIciRNA) (21, 22). The generation mechanisms of circRNA mainly include back-splicing and exon skipping (23, 24) (**Figure 1**). Back-splicing refers to the process in which certain introns are kept during the splicing of precursor RNA, forming a circular structure; this mechanism plays a key role in the generation of circRNA (25, 26). Exon skipping refers to the process in which certain exons are skipped during splicing, resulting in circular RNA that contains multiple exons (27, 28). Back-splicing is one of the main mechanisms for circRNA generation. In the back-splicing process, the 5’ and 3’ ends of precursor mRNA are connected together through back-splicing, forming a closed circular structure (29, 30). This method differs from traditional linear mRNA splicing; instead, it relies on base pairing between reverse complementary sequences of flanking introns (31). It can also involve the dimerization of RNA-binding proteins (RBPs) (32, 33). Research shows that certain RNA-binding proteins and back-splicing factors like QKI and FUS are key players in making circRNA (34, 35). These factors can recognize specific splicing sites and promote the occurrence of back-splicing reactions. Exon skipping is another mechanism for circRNA generation (36). In this mechanism, certain exons in precursor mRNA are skipped, leading to the direct connection of adjacent exons, forming a circular structure (37, 38). Exon skipping is usually regulated by specific splicing factors which can recognize and bind to exon boundary sequences, which helps skip exons (39). Although there is a certain understanding of the biosynthesis mechanism of circRNA, its specific regulatory mechanisms still require further research.

### 2.2 Biological functions of circRNAs

**Figure 2:**
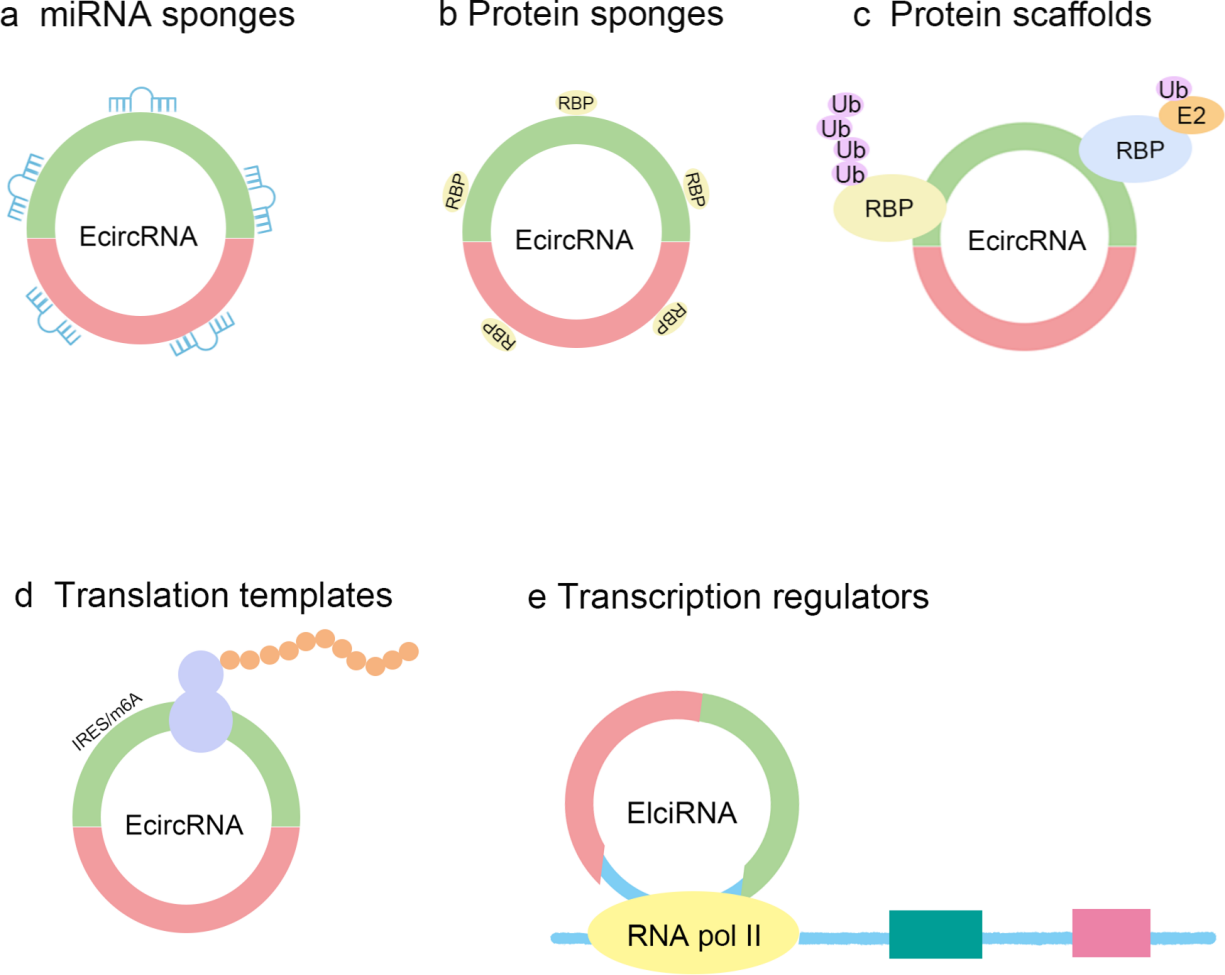
The functions of circRNAs. CircRNA can act as microRNA (miRNA) sponges, thereby affecting the expression of mRNA. CircRNAs can act as protein sponges by competing for shared RNA binding protein binding sites with specific RNA binding proteins (RBPs). CircRNAs can act as protein scaffolds to interact with two RBPs, one of which is usually an enzyme involved in the ubiquitination process. CircRNAs with internal ribosome entry site (IRES) elements or N6-methyladenosines (m6A) modifications can serve as templates for translation into peptide chains. CircRNAs located in the cell nucleus can regulate gene expression. Ub, ubiquitin; E2, Ubiquitin-conjugating enzymes; RNA Pol II, RNA polymerase II.

CircRNA plays various important roles in gene expression regulation (**Figure 2**). Firstly, circRNA can act as a sponge for microRNA (miRNA), preventing miRNA from binding to target mRNA by competitively binding to it, thereby regulating gene expression (9, 40). For example, studies have found that circ_0001490 can regulate the expression of FSTL1 by binding to miR-579-3p, thus affecting the occurrence and development of tuberculosis (41–43). Secondly, circRNA can also interact with RNA-binding proteins (RBPs) to form complexes that regulate the stability, splicing, and translation of mRNA (32, 44). Circular RNA can also serve as a protein scaffold, mediating interactions between two proteins or mRNA, and then forming a complex to exert its function (45, 46). Research has found that upregulated circ-Foxo3 can interact with cyclin-dependent kinase CDK2 and p21 to form a complex, thereby inhibiting the function of CDK2 and suppressing the progression of the cell cycle (47). Furthermore, some circRNAs possess coding functions and can drive their own translation through internal ribosome entry sites (IRES) or N6-methyladenosine (m6A) modification sequences, generating bioactive peptides or proteins (48–50). Research also shown that EIciRNAs mainly exist in the cell nucleus, bind to U1 snRNP, and enhance the transcription of their parent genes (51). It has been reported that the spanning junction open reading frame in circ-FBXW7, propelled by the internal ribosome entry site, encodes an innovative 21-kDa protein (52). This mechanism provides cells with a new pathway for protein synthesis, enriching the diversity of the proteome. CircRNA can form RNA-DNA hybrid chains or R-loops by binding to corresponding DNA sites, leading to the inability to transcribe at the binding site, resulting in exon skipping or transcription termination, thereby producing new splice variants (53, 54). On the other hand, circRNA can also interact with transcription factors to regulate gene transcription (55). These multifunctionalities make circRNA play an important role in cellular physiological activities and have potential regulatory effects in the occurrence and development of various diseases.

## 3 The role of circular RNA in immune response to tuberculosis

### 3.1 Interaction between circRNA and immune cells

CircRNA plays a crucial role in the immune response to tuberculosis, especially in regulating immune cell functions (56). The specific expression of circRNA in different immune cells reflects its key role in immune regulation. Research has found that circRNA can regulate the apoptosis of monocytes, enhance autophagy, and promote the polarization of macrophages, processes that are crucial for the body’s resistance to Mycobacterium tuberculosis infection. Autophagy is an important intracellular degradation mechanism that can eliminate pathogens by enclosing them in autophagosomes and fusing with lysosomes (57). Research indicates that autophagy plays a key role in the clearance of Mtb by macrophages. Macrophages can effectively eliminate Mtb through autophagy, thereby limiting its proliferation within host cells (58). Studies have found that hsa_circ_0002371 is significantly upregulated in tuberculosis patients, and it can promote the expression of hsa-miR-502-5p. ATG16L1 is a target of hsa-miR-502-5p, and the downregulation of ATG16L1 by hsa-miR-502-5p inhibits autophagy in macrophages, promoting the growth of intracellular Mtb (59). Research revealed that the down-regulation of hsa_circ_0045474 triggered macrophage autophagy in tuberculosis through the miR-582-5p/TNKS2 pathway (60). Macrophages are important immune cells that can polarize into different subtypes depending on the microenvironment, mainly including M1 and M2 types. M1 macrophages are generally considered pro-inflammatory, capable of producing a large number of pro-inflammatory cytokines such as tumor necrosis factor α (TNF-α), interleukin-1β (IL-1β), and interferon-γ (IFN-γ) to combat pathogens (61). These cells exert their immune effects by enhancing antigen presentation and activating T cells. In contrast, M2 macrophages mainly participate in anti-inflammatory responses and tissue repair, secreting anti-inflammatory factors such as IL-10 and transforming growth factor β (TGF-β), and promoting wound healing and tissue regeneration (62). The balance between these two polarized states is crucial for maintaining immune homeostasis in the body. In Mtb infection, the polarization state of macrophages significantly affects the outcome of the infection. Mtb can influence macrophage polarization through various mechanisms, thereby affecting the host’s immune response and the progression of the infection (63). Studies have shown that after Mtb infection, macrophages polarize towards the M2 type, inhibiting the pro-inflammatory response of M1 type, a process that may be closely related to bacterial survival and proliferation (64). Research indicates that hsa_circ_0003528 is elevated in tuberculosis patients, and this circular RNA promotes the transition of macrophages from M1 to M2 type by binding to miR324-5p, miR-224-5p, and miR-488-5p (65). Studies have also shown that circTRAPPC6B is a downregulated circular RNA in tuberculosis, which specifically induces the expression of IL-6 and IL-1β by targeting miR-892c-3p, thereby promoting the transition of macrophages from M2 to M1 type, enhancing the host’s clearance of Mtb (14). For instance, research has revealed that circRNA-0003528 upregulates macrophage polarization associated with Mycobacterium tuberculosis by downregulating miR-224-5p, miR-324-5p, and miR-488-5p, and upregulating CTLA4 (65). After Mtb infection, host cells activate multiple signaling pathways to respond to the infection. The main pathways of apoptosis include the intrinsic pathway and the extrinsic pathway. The intrinsic pathway primarily involves mitochondrial dysfunction, leading to the release of cytochrome C, which in turn activates members of the caspase family, such as caspase-3 and caspase-9, thereby triggering apoptosis. The extrinsic pathway is mediated by death receptors (such as Fas and TNF receptors), activating downstream caspases to promote cell death (66). Mtb regulates the apoptosis of host cells through its specific pathogenic factors. For example, the Rv3435c gene of Mtb has been found to inhibit apoptosis in host cells and enhance its survival within the host by suppressing the secretion of inflammatory factors (67). These mechanisms indicate that Mtb regulates the apoptosis of host cells through a complex signaling network to promote its own survival and dissemination. In active tuberculosis, circAGFG1 promotes autophagy and diminishes apoptosis through the miRNA-1257/Notch pathway (68). Additionally, circRNA can also interact with RNA-binding proteins, affecting the activation and function of immune cells (69). For instance, some circRNAs have been found to bind to ribosomes, regulating protein translation and thereby influencing immune cell functions (70). Therefore, circRNA plays unique biological functions in different types of immune cells, further suggesting its potential as a therapeutic target.

### 3.2 Role of circRNA in regulating inflammatory response

CircRNA also plays an important role in regulating inflammatory responses. The inflammatory response is one of the key mechanisms by which the host combats Mycobacterium tuberculosis infection, and circRNA plays multiple roles in this process (71). Studies have shown that circRNA can influence the intensity and duration of the inflammatory response by regulating the expression of inflammation-related genes (41). For example, the overexpression of hsa_circ_0001204 negatively regulates the levels of TLR4, reducing the secretion of pro-inflammatory factors, including interleukin-6 (IL-6), interleukin-1β (IL-1β), and tumor necrosis factor-α (TNF-α) (72). For example, certain circRNAs can upregulate the expression of inflammation-related cytokines by inhibiting the function of miRNAs through binding (73). Research has found that circ-ZNF277 promotes the secretion of inflammatory cytokines IL-1β, IL-6, and TNF-α through the circ-ZNF277/miR-378d/Rab10 axis, thereby inhibiting the survival of Mtb in THP-1 cells (74). Furthermore, circRNA can directly regulate gene transcription by interacting with transcription factors. In tuberculosis, research has found that certain circRNAs can regulate the production of cytokines, thereby affecting the progression of the inflammatory response (75). For instance, circRNA can influence the release of inflammatory factors by regulating the NF-κB signaling pathway (76). These findings suggest that circRNA plays a significant role in regulating inflammatory responses and may become a new target for future tuberculosis treatments.

### 3.3 Functions of circRNA in host immune evasion

Mycobacterium tuberculosis evades host immune surveillance through various mechanisms, and circRNA plays an important role in this process. Studies have shown that circRNA can promote the immune evasion of Mtb by regulating the function of host immune cells. For example, circRNA alters the host’s immune response to Mtb by affecting the state of macrophages and the secretion of cytokines, thereby promoting the persistence of the infection (59, 76). CircAGFG1 enhances the autophagic capacity of macrophages infected with Mtb by regulating the miRNA-1257/Notch signaling pathway, reducing the rate of cell apoptosis, thereby promoting the survival and proliferation of Mtb (68). In addition, studies have found that circRNA_SLC8A1 inhibits the expression of miR-20b-5p, thereby upregulating the expression of SQSTM1/p62 and activating the NF-κB signaling pathway to promote the survival of macrophages infected with Mtb (76). These findings not only reveal the potential role of circRNA in the immune evasion of tuberculosis but also provide new ideas for developing novel immunotherapy strategies against Mtb. Overall, the mechanisms of circular RNA in tuberculosis are multifaceted and context-dependent. They involve complex interactions with miRNAs, regulation of autophagy, and immune responses, among others. As research in this field continues to deepen, the understanding of the specific mechanisms of circular RNA will become more comprehensive.

## 4 CircRNA as biomarkers for tuberculosis

### 4.1 The potential of circRNA in diagnosis of tuberculosis

CircRNA is a type of stable non-coding RNA, and its unique circular structure makes it resistant to degradation by nucleases within cells. This stability results in a higher abundance of circRNA in body fluids (such as serum and saliva), making it an ideal non-invasive biomarker (77, 78). Research shows that the expression of circRNA_051778 is significantly different in malignant pleural effusion associated with lung adenocarcinoma and tuberculous pleural effusion, suggesting that it may play a role in the differential diagnosis between lung cancer and tuberculosis (79). In recent years, researchers have developed various methods for detecting circRNA, including quantitative real-time PCR (qRT-PCR), RNA sequencing, and microarray technology (17, 80–82). These methods not only efficiently detect the expression levels of circRNA but also provide expression profile information in different disease states (83, 84). A study screened serum samples from patients with active tuberculosis using circRNA microarrays and found that circRNA_051239, circRNA_029965, and circRNA_404022 were significantly upregulated in patients with active tuberculosis, while these circRNAs had lower expression levels in serum samples from healthy volunteers, suggesting that they may serve as diagnostic biomarkers for tuberculosis (85). A study using circRNA microarray analysis found that specific circRNA is significantly upregulated in the serum of patients with active tuberculosis, and ROC curve analysis demonstrated its potential as a diagnostic marker for tuberculosis (77, 86, 87). We summarized the circRNAs related to tuberculosis regulation recorded after 2020 in **Table 1**. For instance, hsa_circ_0028883 is significantly more expressed in patients with active tuberculosis compared to healthy controls, and ROC curve analysis indicates its high diagnostic value (88). Investigations indicated that hsa_circ_0002371 was markedly elevated in the PBMCs of ATB patients in comparison to HC. The hsa_circ_0002371/hsa-miR-502-5p/ATG16L1 pathway facilitated the persistence of intracellular Mtb and suppressed autophagy in macrophages (59). This implies that hsa_circ_0002371 might serve as a promising diagnostic biomarker. In addition, combining several circRNAs as biomarkers can enhance diagnostic value. For example, the plasma level of hsa_circ_0001204 can distinguish tuberculosis from healthy controls with a sensitivity of 73.10% and a specificity of 92.50%, with an area under the ROC curve (AUC) of 0.871 (95% CI: 0.827-0.916); while hsa_circ_0001747 can distinguish tuberculosis from healthy controls with a sensitivity of 71.03% and a specificity of 82.50%, with an AUC of 0.830 (95% CI: 0.780-0.880). When hsa_circ_0001204 and hsa_circ_0001747 are used in combination, the AUC increases to 0.928 (95% CI: 0.897-0.960; sensitivity = 86.21%, specificity = 89.17%) (89). In addition, apart from distinguishing between tuberculosis patients and healthy controls, circRNAs also show great potential in diagnosing drug-resistant tuberculosis. Studies have found that the level of circRNA_051239 is significantly elevated in the drug-resistant group, revealing a marker that can be used to differentiate between drug-resistant and sensitive tuberculosis patients (85). These findings indicate that circRNA has significant application potential in the early diagnosis and drug resistance management of tuberculosis. In addition, researchers are able to further explore the functions of circRNA in tuberculosis and its potential applications as a biomarker by establishing circRNA regulatory networks, highlighting the importance of circRNA in disease monitoring and clinical applications (90). These findings provide a solid foundation for the clinical application of circRNA and promote the exploration of early diagnosis for tuberculosis.

**Table 1:** Empirical investigation into the role of circular RNA in tuberculosis.

Circular RNA	Dysregulation	Sample	Method	Function	Ref
hsa_circ_0002371	Up	PBMCs	qRT-PCR	diagnostic biomarker and therapeutic target	(59)
circRNA_SLC8A1	Up	Blood	qRT-PCR	promotes the survival of mtb	(76)
hsa_circ_0082152	Up	Serum	qRT-PCR	promote anti-tuberculosis drug-induced liver injury	(94)
hsa_circ_0007460	Up	Blood	qRT-PCR	diagnostic biomarker and promotes the survival of mtb	(15)
circTRAPPC6B	Down	PBMCs	qRT-PCR	Induced macrophages from M2 to M1	(14)
hsa_circ_0001204	Down	Serum	qRT-PCR	modulates inflammatory response of macrophages	(72)
circRNA-0003528	Up	Monocytes	qRT-PCR	associated macrophage polarization	(65)
hsa_circ_0045474	Down	PBMCs	qRT-PCR	induces macrophage autophagy	(60)
circ-WDR27	Down	PBMCs	qRT-PCR	regulates mycobacterial vitality and secretion of cytokines	(75)
circMARS	Up	Serum	qRT-PCR	a potential biomarker for ADLI diagnosis in TB patients	(95)
hsa_circ_0093884	Down	PBMCs	qRT-PCR	ameliorates hepatocyte	(93)
circ_0001490	Down	Serum	qRT-PCR	inflammation modulates the survival of mycobacteria	(41)
circTRAPPC6B	Down	PBMCs	qRT-PCR	inducing autophagy in macrophages	(92)
circRNA_029965	Up	Serum	Microarray	diagnostic biomarker for TB	(85)
circRNA_051239	Up	Serum	Microarray	diagnostic biomarker for TB	(85)
circRNA_404022	Up	Serum	Microarray	diagnostic biomarker for TB	(85)
hsa_circ_0001380	Down	PBMCs	qRT-PCR	a potential diagnostic biomarker for active tuberculosis	(114)
circAGFGI	Up	Alveolar macrophages	flow cytometry	Enhances autophagy and diminishes apoptosis	(68)
hsa_circ_0028883	Up	PBMCs	qRT-PCR	diagnostic biomarker in active tuberculosis	(88)

### 4.2 The correlation of circRNA with clinical features of tuberculosis

CircRNA is not only potential in the diagnosis of tuberculosis but is also closely related to the clinical features of the disease. Research shows that circRNA plays an important regulatory role in the pathological process of tuberculosis (14, 91). The expression levels of certain circRNAs are closely associated with the severity of the disease, treatment response, and prognosis in tuberculosis patients (92–94). Research has found that the levels of hsa_circ_0043497 and hsa_circ_0001204 in newly diagnosed tuberculosis patients return to normal levels after completing anti-tuberculosis treatment and achieving negative sputum smear for acid-fast bacilli (91). For instance, circMARS is significantly upregulated in patients with anti-tuberculosis drug-induced liver injury (ADLI), and its expression level is positively correlated with the severity of liver damage (94, 95). In clinical practice, based on pulmonary radiographic images, ATB patients can be classified into three severity levels: mild, moderate, and advanced disease. A severity classification was performed on 40 cases of active tuberculosis patients, with each patient receiving a radiological severity score (RSS). The correlation between circRNAs levels and RSS was then analyzed, revealing that hsa_circRNA_001937, hsa_circRNA_009024 and hsa_circRNA_102101 are associated with RSS (84). Furthermore, by constructing a circRNA-miRNA-mRNA competitive endogenous RNA (ceRNA) network, researchers have found that some differentially expressed circRNAs can influence the treatment response of tuberculosis by regulating the expression of key genes (96, 97). These findings indicate that circRNA can serve not only as diagnostic markers for tuberculosis but also as important indicators for assessing disease severity and treatment efficacy, providing new ideas for personalized treatment of tuberculosis.

### 4.3 CircRNA and other biomarkers

miRNAs are an important class of non-coding RNAs that participate in the regulation of gene expression and play a key role in various biological processes. In tuberculosis, the expression patterns of miRNAs undergo significant changes, which may closely relate to the host’s immune response to Mycobacterium tuberculosis (98). Studies have found that specific miRNAs are either upregulated or downregulated in tuberculosis patients, and these changes may reflect the state of infection and the pathological process. For example, miRNAs such as miR-155 and miR-146a show potential for regulating the host’s immune response during tuberculosis infection and may serve as biomarkers for early diagnosis and monitoring of tuberculosis progression (99). Long non-coding RNAs (lncRNAs) are RNA molecules longer than 200 nucleotides. They do not encode proteins but play important roles in regulating gene expression. The expression patterns of lncRNAs are often tissue-specific and developmentally stage-specific, resulting in significant differences in their expression levels across various physiological and pathological conditions. Research has found that lncRNA AC007128.1 is significantly elevated in patients with active tuberculosis and is associated with the clinical characteristics of the patients, suggesting it might play a role in the onset and progression of tuberculosis (100). Using proteomics techniques, researchers have identified a series of tuberculosis-related proteins that show specific expression patterns during infection. For example, antigens such as CFP-10 and ESAT-6 have been extensively studied as potential diagnostic markers, playing a key role in the immune response of infected individuals (101). Metabolites are getting more attention as biomarkers in tuberculosis research. Advances in metabolomics have enabled researchers to identify specific metabolites associated with tuberculosis, which may reflect the host’s physiological state and pathological changes during infection. Studies indicate that certain amino acids and lipid metabolites are significantly elevated or reduced in TB patients, potentially correlating with disease activity and severity (102). CircRNAs are emerging biomarkers that have shown superior sensitivity and specificity in multiple studies. CircRNAs also demonstrate good sensitivity in the early diagnosis of tuberculosis, effectively regulating the host’s immune response and slowing down the apoptosis of monocytes, thereby enhancing resistance to Mycobacterium tuberculosis. Compared to other biomarkers, circRNAs have greater potential for clinical diagnosis due to their stable circular structure, which allows them to persist in blood and other bodily fluids for extended periods. Research indicates that circRNAs show promising prospects for clinical applications in the early diagnosis of diseases like liver cancer and tuberculosis (103). They also exhibit potential in other conditions, such as diabetic nephropathy and cardiovascular diseases (104). The detection methods for circRNAs are relatively simple, providing a non-invasive means of testing for clinical use. With the development of molecular biology techniques, circRNA detection technologies are gradually maturing, demonstrating high adaptability and flexibility for application in various clinical settings. Furthermore, circRNAs are stable and less likely to degrade during transport and storage, further reducing the economic burden during the detection process.

Despite the immense potential of circRNAs in tuberculosis research, future studies still face a lot of challenges. First, the functions and mechanisms of circRNAs have not been fully elucidated, particularly their specific roles in the host immune response require further in-depth investigation. Second, overcoming technical and standardization issues is necessary for the clinical application of circRNAs as biomarkers. Although several studies have demonstrated the potential value of circRNAs, the absence of large-scale clinical trials holds back their use in real-world diagnosis. The impact of individual differences also raises questions about the universality of circRNAs as biomarkers. To enhance the clinical application value of circRNAs, standardized detection processes and guidelines need to be set up. For instance, traditional methods like RNA sequencing and reverse transcription quantitative PCR (RT-qPCR) have some limitations when it comes to validating circRNAs, as they can easily produce false-positive signals; therefore, steps such as nuclease R treatment should be incorporated into experimental design to ensure the accuracy of results (105). Multi-center collaboration and data sharing are important directions for advancing circRNA research. By establishing cross-institutional collaborative networks, resources and data from different research centers can be integrated to enhance the scale and efficiency of research. For example, the data warehouse project in the Netherlands successfully integrated electronic health records of critically ill COVID-19 patients from different hospitals, providing a rich data foundation for clinical research (106). This collaborative model not only accelerates the discovery and validation of new biomarkers but also helps quickly turn research findings into practical applications. Additionally, the design and implementation of clinical trials are crucial issues that need to fully consider the safety and efficacy of circRNAs. Regulatory and ethical issues must also be properly addressed to ensure that the clinical application of circRNAs meets relevant standards and requirements.

These challenges also create opportunities for researchers, promoting the integration of multi-omics studies to explore the interactions between circRNAs and the host genetic background, thereby providing new ideas and methods for the early diagnosis and personalized treatment of tuberculosis. For instance, polymorphisms in certain genes are associated with patients’ responses to TB treatment, providing a theoretical basis for personalized therapy (107). Through interdisciplinary collaboration, future research is likely to make breakthroughs in the discovery of biomarkers for tuberculosis and the formulation of personalized treatment strategies.

## 5 Application prospects of circRNA in tuberculosis treatment

### 5.1 The possibility of circRNA as a therapeutic target

The application prospects of circRNA in tuberculosis treatment mainly lie in its potential as a therapeutic target. Research indicates that circRNA plays an important regulatory role during Mycobacterium tuberculosis infection. For example, certain circRNAs can enhance anti-tuberculosis effects by modulating the host immune response, such as enhancing autophagy and promoting macrophage polarization (59, 75). These functions make circRNA a potential therapeutic target; by targeting these circRNAs, the host immune response can be regulated, thereby improving the efficacy of anti-tuberculosis treatment (108). Additionally, the stability and specific expression patterns of circRNA make its application in therapy more feasible (109). In the treatment of tuberculosis, strategies targeting circRNA have started to gain traction. The main methods for targeting circRNA include gene editing technologies, RNA interference (RNAi), and antisense oligonucleotides (ASO). Gene editing methods like the CRISPR/Cas system can precisely target specific circRNA, which helps regulate its expression and affects related signaling pathways and biological functions (110). RNAi technology silences the target circRNA through small interfering RNA (siRNA) or short hairpin RNA (shRNA) (111). It is noteworthy that circRNA has complex biological functions, and targeted therapy must comprehensively consider the role of circRNA in tuberculosis to ensure the effectiveness and safety of the treatment. A major challenge in treating tuberculosis is drug resistance, especially with the emergence of multidrug-resistant (MDR-TB) and extensively drug-resistant (XDR-TB) strains, making treatment more complicated. Studies have shown that circRNA plays a role in managing cancer drug resistance; for example, circ_0007823 has been found to be associated with cisplatin resistance in triple-negative breast cancer, where its overexpression weakens cisplatin resistance through the miR-182-5p-FOXO1 axis (112). In tuberculosis, some studies have found that certain circRNA are upregulated in drug-resistant strains of tuberculosis infection, suggesting that targeting circRNA could be a new strategy to tackle drug resistance in tuberculosis. In summary, targeting circRNA offers new treatment ideas for managing drug-resistant tuberculosis. By regulating the expression and function of circRNA, we could effectively reverse drug resistance mechanisms and enhance the sensitivity of Mycobacterium tuberculosis to treatment. Research in this area is still ongoing, and it’s expected to lead to better treatment options for patients with drug-resistant tuberculosis down the line. Although current research on circRNA in tuberculosis treatment is still in its preliminary stages, studies have shown its significant potential in regulating gene expression and cellular functions (113). In the future, in-depth research on the functional mechanisms of circRNA is expected to lead to the development of circRNA-based targeted therapeutic strategies, providing new ideas for tuberculosis treatment.

### 5.2 The role of circRNA in novel drug development

The role of circRNA in novel drug development is also receiving considerable attention. Due to the specific expression and stability of circRNA in tuberculosis, they are considered ideal candidate molecules for developing new anti-tuberculosis drugs (90). For instance, certain circRNAs can act as miRNA sponges, regulating miRNA functions and thereby affecting gene expression and cellular behavior (14). This regulatory mechanism provides new avenues for developing circRNA-based drugs. Additionally, circRNA can interact with RNA-binding proteins to regulate protein localization and function, thus influencing physiological processes in cells (93). These characteristics make circRNA have significant application prospects in novel drug development. Through in-depth research on the biological functions and mechanisms of circRNA, it is hoped that innovative circRNA-based drugs can be developed, providing new options for tuberculosis treatment. In summary, circRNA has broad application prospects in tuberculosis treatment, and future research will further reveal its potential and mechanisms in therapy, providing new strategies and ideas for the prevention and control of tuberculosis.

## 6 Conclusion

The progress of circRNA research in tuberculosis provides us with new perspectives, revealing its potential applications in pathological mechanisms, diagnosis, and treatment. Studies indicate that circRNA not only exhibits specific expression profile changes in Mycobacterium tuberculosis infection but also plays an important role in the host immune response. These findings emphasize the potential of circRNA as biomarkers, which may provide new evidence for the early diagnosis of tuberculosis. Furthermore, the role of circRNA in regulating inflammatory responses and immune cell interactions further expands our understanding of the immune mechanisms of tuberculosis.

However, despite the encouraging results of current research, there are still many challenges in the clinical application and mechanistic exploration of circRNA. Different viewpoints and findings among studies may vary due to differences in experimental design, sample selection, and technical methods, so we need to be cautious in interpreting the biological functions of circRNA. Future research should focus on establishing standardized experimental methods to better compare and validate different research results. At the same time, in-depth exploration of the specific mechanisms of circRNA in tuberculosis will provide a more solid foundation for its potential as a therapeutic target. Research on circRNA should focus on its feasibility in clinical applications, especially in the formulation of personalized treatment strategies. By integrating different research perspectives and balancing various findings, we hope to promote the practical application of circRNA in the prevention and control of tuberculosis, ultimately providing new ideas and strategies for the global control and elimination of tuberculosis.
